# Recent Advancements of Polyaniline/Metal Organic Framework (PANI/MOF) Composite Electrodes for Supercapacitor Applications: A Critical Review

**DOI:** 10.3390/nano12091511

**Published:** 2022-04-29

**Authors:** Rajangam Vinodh, Rajendran Suresh Babu, Sangaraju Sambasivam, Chandu V. V. Muralee Gopi, Salem Alzahmi, Hee-Je Kim, Ana Lucia Ferreira de Barros, Ihab M. Obaidat

**Affiliations:** 1 Department of Electronics Engineering, Pusan National University, Busan 46241, Korea; vinoth6482@gmail.com; 2 Laboratory of Experimental and Applied Physics, Centro Federal de Educação Tecnológica Celso suckow da Fonesca, Av. Maracanã Campus 229, Rio de Janeiro 20271-110, Brazil; ryesbabu@gmail.com (R.S.B.); ana.barros@cefet-rj.br (A.L.F.d.B.); 3 Department of Physics, United Arab Emirates University, Al Ain P.O. Box 15551, United Arab Emirates; sambaphy@gmail.com; 4 Department of Electrical Engineering, University of Sharjah, Sharjah P.O. Box 27272, United Arab Emirates; naga5673@gmail.com; 5 Department of Chemical & Petroleum Engineering, United Arab Emirates University, Al Ain P.O. Box 15551, United Arab Emirates; 6 National Water and Energy Center, United Arab Emirates University, Al Ain P.O. Box 15551, United Arab Emirates; 7 Department of Electrical and Computer Engineering, Pusan National University, Busan 46241, Korea

**Keywords:** polyaniline, metal–organic framework, supercapacitors, energy density, specific capacitance, stability

## Abstract

Supercapacitors (SCs), also known as ultracapacitors, should be one of the most promising contenders for meeting the needs of human viable growth owing to their advantages: for example, excellent capacitance and rate efficiency, extended durability, and cheap materials price. Supercapacitor research on electrode materials is significant because it plays a vital part in the performance of SCs. Polyaniline (PANI) is an exceptional candidate for energy-storage applications owing to its tunable structure, multiple oxidation/reduction reactions, cheap price, environmental stability, and ease of handling. With their exceptional morphology, suitable functional linkers, metal sites, and high specific surface area, metal–organic frameworks (MOFs) are outstanding materials for electrodes fabrication in electrochemical energy storage systems. The combination of PANI and MOF (PANI/MOF composites) as electrode materials demonstrates additional benefits, which are worthy of exploration. The positive impacts of the two various electrode materials can improve the resultant electrochemical performances. Recently, these kinds of conducting polymers with MOFs composites are predicted to become the next-generation electrode materials for the development of efficient and well-organized SCs. The recent achievements in the use of PANI/MOFs-based electrode materials for supercapacitor applications are critically reviewed in this paper. Furthermore, we discuss the existing issues with PANI/MOF composites and their analogues in the field of supercapacitor electrodes in addition to potential future improvements.

## 1. Introduction

In recent times, the energy crisis has resurfaced as a severe social issue that is stifling growth and eventually endangering human survival [1]. Due to the economic surge, global consumption for sustainable and alternative energy resources is growing relentlessly alongside a vigorous worldwide upsurge in concern regarding ecological issues such as global warming, inappropriate climate change (including wildfire, melting glaciers, floods, drought, increasing in ocean level), and most important, the sustainability of oil reserves. Energy storage and conversion technologies that are renewable, safe, clean, and long-lasting have become a hot research topic [2,3,4,5]. Advances in the development of clean, renewable, safe, and practical energy storage systems such as batteries and supercapacitors and fuel cells [6,7,8,9,10] have attracted widespread interest from the scientific community. In recent times, electrochemical energy storage devices have gained considerable attention due to their higher energy efficiency and ecological power systems [11,12,13]. SCs are presently found in consumer electronics, tools, power supply, voltage stabilization, microgrid, renewable energy storage, energy harvesting, streetlights, medical applications, military, and automotive applications [14,15,16,17,18,19]. Recently, a commercial corporation offered a 48 V ultra-capacitor module with 1,000,000 duty cycles or a ten-year DC life and 48 V DC working voltage [20]. The modules were engineered explicitly for hybrid bus and construction equipment to provide cost-effective solutions. Furthermore, Maxwell Technologies and LS Mtron Corporations offered different voltage module SCs with a high cycle life and 48 V DC working voltage [21].

**Figure 1:**
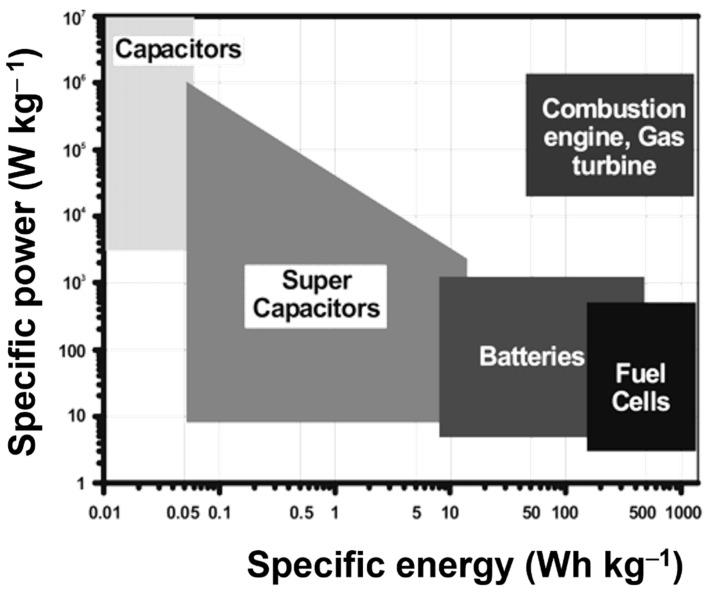
Ragone plot for the various electrochemical energy storage devices. Reproduced with permission from [22]. Copyright 2004 American Chemical Society.

Figure 1 shows a Ragone plot of the specific energy (Wh kg^−1^) versus the specific power (W kg^−1^), which is used to evaluate the performance of various energy storage technologies. The logarithmic scale of both vertical and horizontal axes and the performances of different systems can be accessibly evaluated. The first version of this type of graph was used to compare the performance of batteries. However, it is appropriate for comparing any kind of energy storage systems. The fuel cells are high-energy-density devices, while SCs are high-power-density devices, as shown in this diagram. Batteries have intermediate power (*P*_d_) and energy (*E*_d_) densities. Furthermore, no electrochemical device can compete with an internal combustion engine, as shown in Figure 1. Hence, to compete with the combustion engine, the *E*_d_ and *P*_d_ values of electrochemical systems must increase [22]. Batteries can deliver specific energy between 150 and 500 Wh kg^−1^ [23,24,25,26,27,28] but are limited to their poor specific power because of sluggish electron and ion transport at high rates. To sustain a higher energy output, their discharge time is usually more than 600 s or even 60 min. In contrast, electric double-layer capacitors (EDLCs) which are characterized by high specific power can completely release their energy within less than 10 s, providing a power output between 10- and 20- kW kg^−1^ [29,30,31,32]. The specific energy and specific power based on the recently reported work with respect to the supercapacitors has been presented in Table S1 (please refer to the supporting information section).

Unlike fuel cells and batteries, SCs are electrochemical capacitors that store electric charges in electric double layers that form at the electrode–electrolyte interface. SCs are presently found in consumer electronics, memory storage devices, and industrial power/energy organization systems. The SC is composed of high surface-area electrodes (such as anode and cathode), an electrolyte (for example, aqueous medium/organic medium), and a separator (which avoids short circuits among anode and cathode). The electrode is an important element that controls the performance of the SC. The construction of ultrahigh performance SC electrodes includes various serious characteristics such as high specific surface area, extraordinary conductivity, stability based on temperature, optimizing the distribution of pore size, appropriate processing, adequate corrosive resistance, and cost efficiency [33,34,35,36,37,38]. Hence, the selection of appropriate materials and optimizing the electrode design are vital approaches to convert SCs into more energy-efficient energy storage devices than secondary ion batteries [39,40,41,42,43,44,45].

### 1.1. Classification of Supercapacitors

**Figure 2:**
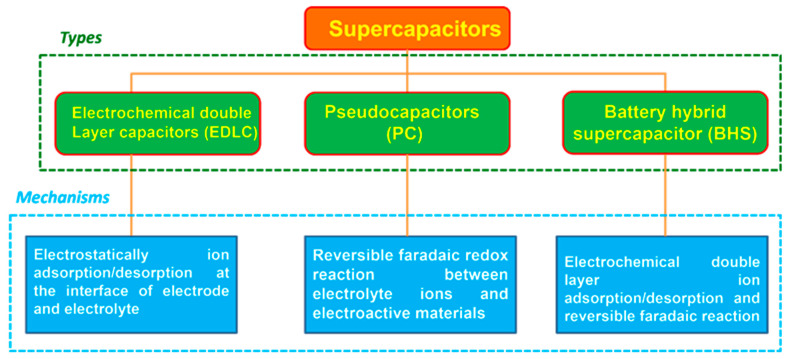
Types of supercapacitors and its mechanism.

Supercapacitors are divided into three kinds, namely an electric double layer capacitor (EDLC), pseudocapacitor (PC), and battery hybrid supercapacitor (BHS) based on the mechanism of energy storage, as illustrated in Figure 2.

#### 1.1.1. EDLCs

EDLCs include two separate carbon-based materials employed as electrodes: an electrolyte as well as a separator. EDLC can store the charges electrostatically, which is a non-Faradaic process that does not require the transfer of charges between the electrode/electrolyte interfaces [46]. EDLCs use the electric double-layer model for energy storage mechanism. The electrons migrate from the anode to the cathode via the external loop during the charging process, with anions moving toward the cathode and cations moving toward the anode in the electrolyte. The electrons and ions flow in opposite directions during the discharging process. The energy storage process is non-Faradaic, and there are no redox reactions, since no charges flow across the electrode–electrolyte contact. Because of the non-Faradaic charge storage mechanism, the volume and morphology of the electrode materials hardly changes, resulting in EDLCs’ extended cycle-life [47,48]. Furthermore, the mechanism of charge storage in EDLCs allows quick energy uptake, delivery, and exceptional power output. EDLCs have the potential to withstand millions of cycles compared to batteries with maximum capacity. In lithium-ion batteries (LIBs), when high potential positive electrodes or graphite negative electrodes are employed, the charging process does not need an electrolyte; this leads to a solid electrolyte intermediate [49]. In general, EDLCs employ carbon electrode materials, such as graphene, activated carbon, nano-architectured carbon, and carbon aerogels, for the accumulation of charge via the reversible adsorption/desorption of ions at the electrode/electrolyte interface [50,51,52]. EDLC materials have been studied extensively owing to their high SSA [53], good electrical conductivity, and excellent mechanical stability [54], but they suffer from a low specific capacitance [55].

#### 1.1.2. PCs

The Faradaic charge-storage mechanism, such as redox reactions, involves the transfer of charge between the electrolyte and electrode. In PCs, when a potential is applied to the electrode material, a redox reaction occurs at the surface of the electrode and electrolyte, which cause the charges to pass through the double layer and results in Faradaic current via the SC cell. When compared to EDLCs, the Faradaic mechanism used in PCs allows for higher specific capacitance and energy density [47,56]. Suitable materials for PCs are thoroughly being explored such as transition metal oxides (TMOs), which provide a relatively high specific capacitance and greater specific energy with a good intrinsic conductivity [57,58], making them exceptional candidates for high-performance SCs. Unfortunately, PCs suffer from inferior cyclic stability performance due to the frequent swelling and shrinking of the polymer chains during the doping/de-doping procedure [59,60,61,62,63]. For TMOs, the major drawback is the low conductivity, which hinders them from reaching the high theoretical specific capacitance value.

#### 1.1.3. Hybrid Supercapacitors

A hybrid supercapacitor is a supercapacitor with asymmetric electrodes, one with electrostatic capacitance, and the other with electrochemical capacitance. The hybrid supercapacitors reached previously unachievable performance characteristics. Furthermore, they combine the great features associated with PCs and EDLCs into one integrated supercapacitor. Although hybrid supercapacitors are less studied compared to EDLCs and pseudocapacitors, efforts are increasing in terms of developing improved hybrid supercapacitors and creating accurate quantifiable models. Developing the high energy density and long-term cycling stability of hybrid supercapacitors has overtaken EDLCs as a class of core SCs [64]. Hybrid supercapacitors are divided into three groups, which differ by their arrangement of electrodes: asymmetric, composite, and battery type.

##### Composite

Carbonaceous materials are mixed with conducting polymers and (or) metal oxides to fabricate composite electrodes, demonstrating that a single electrode may store energy in both chemical and physical modes. There are two types of composites: (i) binary composites—the electrode material is combination of two materials, and (ii) ternary composites—the electrode material comprises three different materials [47].

##### Asymmetric

Asymmetric-type supercapacitors are combining the process of Faradaic and non-Faradaic by connecting the electrodes of the pseudocapacitor with EDLCs. In this manner, the conducting polymer or metal oxide is employed as the cathode and the carbon-based material is employed as the anode [47].

##### Battery Type

The battery-type supercapacitors are a one-of-a-kind integration of a battery and SC electrode materials. This design demonstrates the requirements for greater power density batteries and greater energy density capacitors by integrating SC and battery characteristics in a single cell to achieve both battery and SC properties. Battery-type materials have been widely developed and studied for hybrid supercapacitors because of their richer Faradaic reactions and higher energy density. However, the redox reactions that emerged in bulk materials and the phase transformation process may result in sluggish kinetics and poor rate capability, which need to be improved further [65]. There is a typical feature in electrochemical tests for the battery-type electrode materials: they possess obvious redox peaks and a nonlinear potential platform, while those from capacitive and pseudo-capacitive materials are quite different [66]. Therefore, the specific capacity with a unit of C/g (mAh/g) instead of F/g for specific capacitance is employed to express the capability of charge storage for the battery-grade materials. Binary transition metal oxides (BTMOs) such as NiCo_2_O_4_ [67], MgCo_2_O_4_ [68], CuCo_2_O_4_ [69], and ZnCo_2_O_4_ [70] have been reported as battery-grade electrode materials. In their crystal structure, some metals can provide variable oxide states for plenty of redox reactions, and thus, the specific capacity is expected to be enhanced [71].

## 2. Conducting Polymers (CPs)

Owing to their unique features, CPs have been regarded, to date, as reliable and excellent electrode materials for pseudocapacitors. Several CPs, for example PANI, polypyrrole (PPy), and polythiophene (PTh), are important for energy-storage applications. These materials have variety of advantages, including excellent conductivity, flexibility, low cost, and ease of preparation [72]. Furthermore, many scientists have investigated the CPs electrodes for their electrochemical performances and attempted to enhance their properties in several ways. In this section, we evaluate the current state of research on pure PANI-based electrode materials for supercapacitor applications.

### PANI

PANI is an excellent CP, which can be polymerized with monomer of aniline by various techniques, and it has many advantages because of its facile preparation, easy acid/base chemistry (insertion/desertion), and ecological sustainability [73]. PANI has turned into one of the most efficient materials for PC electrodes. The morphology of PANI nanostructures has a significant impact on their electrochemical performances; therefore, it is very important to employ a suitable and high-efficacy preparation technique to produce PANI with the appropriate nanostructure. Indeed, the chemical or electrochemical polymerization of PANI is rather simple. PANI prefers to form nanofibers in an aqueous solution during chemical oxidative polymerization [74], and there are several polymerization methods for obtaining PANI nanostructures [75,76,77]. Interfacial polymerization is quite simple and one of the least expensive and general methods to prepared PANI.

Sivakkumar et al. [78] used an interfacial polymerization process to make PANI nanofibers. Their electrochemical characteristics were evaluated in a two-electrode cell configuration with an aqueous electrolyte where the device was reported to exhibit extraordinary specific capacitance of 554 F g^−1^ at 1 A g^−1^. However, it showed very poor cyclic stability where the initial value of capacitance declined sharply. The theoretical and experimental capacitances of PANI in sulfuric acid medium were reported by Li et al. [79]. Because the specific capacitance of PANI depends on both the conductivity of PANI and the diffusion of counter-anions, the PANI theoretical capacitance value is approximately 2000 F g^−1^, whereas the experimental values calculated by various methods are less than the theoretical value.

In conclusion, a large quantity of bare PANI electrode material has been investigated for the use in supercapacitors, but its electrochemical performance, notably cycle stability, did not meet commercial application criteria. The poor cyclability of the supercapacitors results in a rapid decrease in its specific capacitance and thus a shorter cycle-life. Hence, for improving the performance of supercapacitors, the scientific community has attempted to mix PANI with carbonaceous materials, metal oxides, metal hexacyanoferrates and/or MOFs to produce various PANI-based composites, particularly electrochemical characteristics [80,81,82,83,84].

## 3. MOFs

Over the last decade, MOFs, also called coordination polymers, have attracted the attention of materials research. They are constructed as a “node-spacer” of nanosized materials. MOFs contain metal centers (cluster/ions), which are coupled through organic linkers (groups comprising imidazole/carboxyl) to synthesize crystalline, durable, and often very fine porous structures. MOFs exhibit a variety of improvements over the traditional porous materials: for example, rationally designed and highly desirable crystal structures of achievable crystal engineering. Furthermore, the high synthetic flexibility of MOFs with the ease of combining different chemical functionalization leads to engineering MOFs with lightweight organic linkers that result in a high specific surface area and excellent porosity that are inaccessible to traditional materials such as zeolites and porous carbon [85,86,87].

For instance, Vinodh et al. reported on the effect of Co/Zn ratio on the synthesis of zeolitic imidazole frameworks (ZIFs), where it displayed remarkable ability on the specific surface area, crystal structure, pore size, and electrochemical performances [88]. The maximum BET surface area of ZIF with Co/Zn = 0.5 was found to be 1043.65 m^2^ g^−1^. The ZIF with Co/Zn = 0.5 electrode exhibited a specific capacitance maximum of 30 F g^−1^ at a current density of 0.2 A g^−1^. Furthermore, ZIF with a Co/Zn = 0.5 electrode retained 91.7% of its initial capacitance over 2000 GCD cycles.

Although their weak conductivity does not ensure higher specific capacitance, pristine MOFs and their derived structures possess an enhanced quantity of pores, leading to higher specific surface areas, as previously noted [89]. Furthermore, the energy density and power density values are not at the preferred levels. To mitigate such deficiencies, different techniques have been introduced: for example, the MOFs intercalation with CPs such as PANI, PPy, and polyethylene dioxythiophene (PEDOT) [90,91]. The CPs have been developed to synthesize, delivering high pseudocapacitance and excellent stability on the long term. In supercapacitors, the charge storage mechanism of their CP electrodes is Faradaic [91]. Combining CPs and MOFs produced a supercapacitor electrode material with remarkable electrochemical properties. PANI is one of the most extensively utilized CPs for such applications due to its simplistic synthesis, excellent conductivity, and high pseudocapacitance behavior [92].

## 4. PANI/MOF Composite Electrode Material for Supercapacitor Applications

Wang et al. reported the reduction in MOFs bulk resistance with efficient methodology using interweaving MOF crystals into PANI chains that are electrically coated on MOFs [93]. Briefly, cobalt-based MOF crystals (ZIF-67) were deposited on carbon cloth (CC), and then, PANI was electrochemically deposited to provide a flexible porous electrode (PANI-ZIF-67-CC) without changing the MOF primary structure. From the electrochemical examination, the prepared PANI-ZIF-67-CC showed an outstanding areal capacitance of 2146 mF cm^−2^ at the sweep rate of 10 mV s^−1^. Furthermore, a symmetric flexible solid-state supercapacitor (SFSS) was constructed and evaluated.

Xu et al. synthesized a simple stirring method of ZIF-67 and PANI composites (ZIF-67/PANI) [94]. Additionally, sulfur was incorporated into ZIF-67/PANI using sulfurization (Co_3_S_4_/PANI). The electron transfer process was enhanced by introducing sulfur for its lower electronegativity. The specific capacitance of Co_3_S_4_/PANI achieved was 1106 F g^−1^ at 1 A g^−1^, which is approximately 11 times higher than that of ZIF-67. The constructed asymmetric supercapacitors (ASC) device showed a high energy density of 40.75 Wh kg^−1^ at a specific power of 800 W kg^−1^ and displayed excellent cyclic life. In addition, the fabricated ASC retained 88% of its initial capacitance over 20,000 charge/discharge cycles at a higher current density (5 A g^−1^). Furthermore, the authors stated that the outstanding electrochemical performances suggested that the fabricated electrode could possess virtuous market prospects and could be an appropriate candidate in energy storage fields.

**Figure 3:**
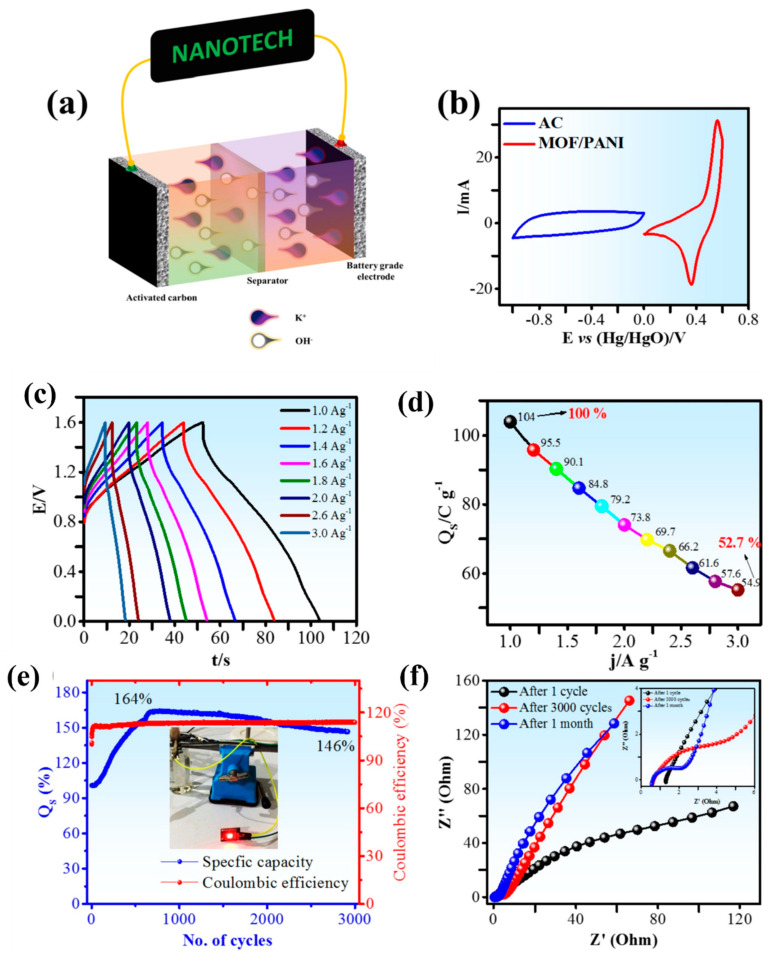
Pictorial illustration of the ASC device, AC//MOF/PANI (**a**); separate CV profiles of AC anode and MOF/PANI cathode (**b**); GCD curves at various densities of current (**c**); specific capacity vs. current density plot (**d**); specific capacity and columbic efficiency plot (**e**); and EIS of before stability test, after 3000 GCD cycles and one month later (**f**). Reproduced with the permission from [95]. Copyright 2020 Elsevier.

Iqbal et al. reported cobalt intercalated in a composite of MOF/PANI for the supercapattery device applications [95]. The ASC supercapattery device (AC//MOF/PANI) was fabricated using the activated carbon (AC) and MOF/PANI as the anode and cathode, respectively (Figure 3a). The working voltage window of the constructed ASC was the combination of voltage windows of both of the electrodes.

Figure 3b shows the cyclic voltammetry (CV) of both MOF/PANI, and AC electrodes that were recorded individually, in a three-electrode compartment to examine the plausible wide voltage window. Furthermore, Figure 3c shows the galvanostatic charge/discharge (GCD) plateaus for the fabricated ASC device. The GCD curves are neither triangular nor humped shapes but have a combination of both shapes, which are in good arrangement with the CV traces. The GCD profiles for the ASC device at various current densities ranging from 1 to 3 A g^−1^ are depicted in Figure 3d between the cut-off window of 0 and 1.6 V. The GCD plateaus at different densities of current are nearly linear (symmetrical) with the minimal ohmic drop indicating a reduction in the internal resistance and excellent rate capability confirming the high columbic efficiencies of the fabricated device. The ASC device showed a specific capacity maximum of 104.5 C g^−1^ at 1 A g^−1^.

Figure 3e exhibits the electrochemical impedance spectroscopy (EIS) examinations which display the finest performance and exceptional electrical conductivity of the supercapattery device. Furthermore, the constructed ASC device delivered outstanding performance with the energy density of 23.2 Wh kg^−1^ with higher power density of 1600 W kg^−1^ at 1 A g^−1^ along with outstanding stability (3000 GCD cycles and endure specific capacity of 146%).

Yao et al. have prepared porous carbon frameworks derived from MOFs (PC-MOFs) as the substrate and deposited PANI via in situ polymerization [96]. The structurally stable porous carbon frameworks derived from MOFs and the homogeneously immobilized conducting PANI nanowires resulted in a PC-MOFs/PANI hybrid electrode with a supreme capacitance of 534.16 F g^−1^ at 0.2 A g^−1^ and an extreme capacitance maintenance of 211% at 2 A g^−1^ after 20,000 GCD curves. In addition, the constructed symmetrical supercapacitors (SSC) resulted in excellent electrochemical performance (specific power of 9.72 μWh cm^−2^) and outstanding cyclability (94.4% at 10,000 cycles), which can be powered with commercial LED.

**Figure 4:**
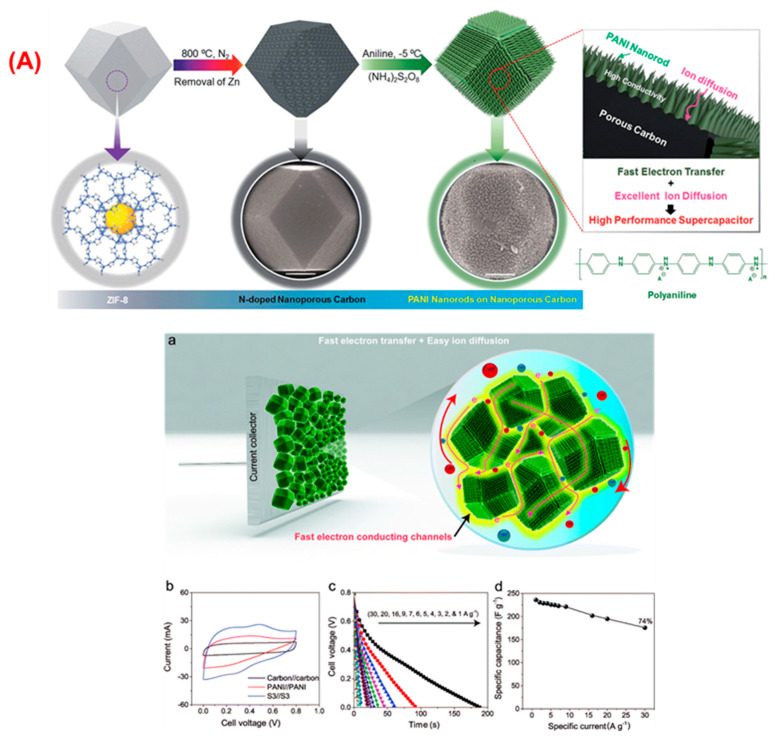
(**A**) Schematic illustration and preparation methodology of the nanoporous carbon/PANI core–shell nanocomposites from the ZIF-8; electrochemical performances, (**a**) distinctive multifaced nanoarchitecture avoids the general issue of stacking caused by one-dimensional CNTs or two-dimensional graphene, which allows ions to penetrate deeper into the material more easily; (**b**) CVs of carbon//carbon, PANI//PANI and carbon–PANI//carbon–PANI (S3), capacitors in 1 M H_2_SO_4_ electrolyte. (**c**) Discharge profile for the S3 capacitor at various current densities; (**d**) plot of specific capacitance vs. current density. Reproduced from [97].

In another prominent work, Salunkhe et al. fabricated SSC based on a core–shell 3D structure consisting of MOF derived nanoporous carbon-PANI composite electrodes [97]. A pictorial representation of the preparation methodology for the achievement of a core–shell structure of nanoporous carbon-PANI nanocomposites is revealed in Figure 4A. This configuration has the advantage of improving the mechanical strength of the polymer without blocking the carbon core’s electronic conductivity as well as providing a direct diffusion path to the core. The unique multifaced nanoarchitecture avoids the general issue of stacking caused by one-dimensional CNTs or two-dimensional graphene, and thus, it allows ions to penetrate deeper into the material more easily.

In addition, the PANI nanorod arrays deliver the ions with simple contact to the carbon core, which lead to the improved interaction of these nanocomposites. In addition, the PANI nanorods provide electrons with rapid conducting routes (electron highways) to attain the current collector surface (Figure 4a). The synthesized composites allowed well-organized electrochemical entry to the electrolyte ions. The comparative CVs of the three different materials are revealed in Figure 4b. The GCD studies were examined at different densities of current ranges from 1 to 30 A g^−1^. As seen in Figure 4c, the GCD plots are linear and without ohmic drop up to 30 A g^−1^. Consequently, a higher value of capacitances (between 300 and 1100 F g^−1^) was attained (Figure 4d). The SSC assembled with this composite material displayed a supreme specific energy of 21 Wh kg^−1^ at a specific power of 12 kW kg^−1^. Approximately 86% of its original specific capacitance was maintained over 20,000 GCD profiles.

Milakin et al. prepared a composite of PANI/Fe-BTC by the in situ polymerization of aniline monomer in the presence of Fe-BTC [98]. The increasing ratio of aniline and Fe-BTC was found to enhance the gravimetric capacitances value of the composite electrode materials, achieving superior capacitance of 346 F g^−1^ at a sweep rate of 20 mV s^−1^. In addition, the enhanced pseudocapacitance behavior and the significantly better reversibility throughout the electrochemical techniques displayed by the prepared composite electrode (PANI/Fe-BTC) compared to virgin PANI could be beneficial for supercapacitor applications.

Wang et al. studied a novel flexible solid-state micro supercapacitor (MSCs) with good specific power, outstanding cyclic stability, and excellent mechanical flexibility [99]. The MSCs were constructed by layer-by-layer electrodeposition of microporous PANI and the MOFs crystals on the substrate of laser-induced graphene. Due to the combined effects of MOFs with higher pore structure and the outstanding conductivity of PANI chains, the resultant MSCs showed layer-dependent capacitance performances, resulting in a very high areal specific capacitance of 719.2 mF cm^−2^ at 0.5 mA cm^−2^. The obtained specific capacitance value was approximately 370 folds better than that of MSCs made by the virgin LIG. Furthermore, the fabricated MSCs retain almost 87.6% of its initial specific capacitance over 6000 GCD curves, illustrating their remarkable cycling stability. In addition, the usage of MSCs for light-emitting diode and their constant mechanical flexibility demonstrate their outstanding potential as electricity for the small and wearable electronics.

Guo et al. developed a high-performance carbonized composite electrode material (Zn-MOF/PANI) from aniline monomer, 8-hydroxyquinoline, and zinc acetate by a facile process for supercapacitor applications [100]. The electrochemical characteristics of the carbonized composite electrode were explored by GCD and CV techniques. The maximum capacitance of 477 F g^−1^ at 1 A g^−1^ was achieved for MOF/PANI composite material.

Shao et al. employed a stable interpenetration polymer network (IPN) structure using extremely stable microscopic MOFs with various synergistic effects to improve the conductivity and electrochemical characteristics, using an efficient approach to grow the molecular chains of PANI in the pores of UiO-66 (PANI/UiO-66) [101]. Furthermore, the prepared composite electrode, PANI/UiO-66, displayed a specific capacitance maximum of 1015 F g^−1^ at 1 A g^−1^. The assembled supercapacitor displayed a promising capacitance of 647 F g^−1^ at a current density of 1 A g^−1^ and an extraordinary cyclic stability (retains almost 91% of its original specific capacitance over 5000 GCD curves). The bending angle test designates that the attained SC was bendable, and only 10% of its original value declined over 800 twisting cycles with 180° (bending angle). Therefore, the authors suggested that the flexible solid-state supercapacitor (FSSC) could be a potential contender in energy storage device.

Liu et al. established a facile and efficient approach to prepare MOFs derived SC by an in situ network of ZIF-67 particles covered by conducting polyaniline [102]. The attained ZIF-67/PANI electrode material possesses an extraordinarily huge porous surface area and excellent electrical conductivity, ensuring an astonishingly superior specific capacity of 1123.65 C g^−1^ (2497 F g^−1^) at 1 A g^−1^ in a three-electrode configuration and a remarkable cycling performance (capacitance retention of 92.3% over 9000 cycles at 5 A g^−1^) for ZIF-67@PANI-2. Furthermore, ZIF-67@PANI-2 displayed a high specific power of 504.72 Wkg^−1^ at a high specific energy of 71.1 Wh kg^−1^ at 1 A g^−1^.

Xu et al. grew leaflike ZIF nanosheets (ZIF-L) into carbon fiber paper (CFP) by a simple single-step immersing technique with the absence of binders and conductive additives [103]. In contrast, three-dimensional ZIF-67 nanoparticles were also employed as electrode materials. The meager intrinsic conductivity and poor capacitance of ZIFs were enhanced by interlacing with polyaniline. The composite CFP/ZIF-L/PANI showed an area capacitance of 730 mF cm^−2^ at 10 mV s^−1^, which is higher than that of CFP/ZIF-67/PANI (608 mF cm^−2^). In addition, the CFP/ZIF-L/PANI electrode maintained 82.6% of its initial specific capacitance over 3000 GCD cycles.

Udayan et al. employed a facile approach to alter ZIF-8 with polyaniline through a precise interfacial polymerization technique to synthesize ZIF-8/PANI nanocomposites [104]. The present methodology evades the accumulation of ZIF-8/PANI, lifts the consumption of active materials, and disclosures additional active sites, thus making it advantageous for simple electron transfer. Owing to its unique multiporous architecture, ZIF-8/PANI had a large specific surface area of 610.8 m^2^ g^−1^, and the ZIF-8/PANI electrode showed a supreme specific capacitance of 395.4 F g^−1^ at a current density of 0.2 A g^−1^. A solid-state ASC constructed with ZIF-8/PANI displayed an excellent performance over a wide operating voltage window from 0 to 2.5 V without non-aqueous electrolytes. It showed a specific areal capacitance of 28.1 mF cm^−2^ at 0.1 mA cm^−2^. The solid-state ASC also displayed a high specific energy (3.2 µW h cm^−2^) and specific power (1.1 mW cm^−2^), remarkable cycling stability, and flexibility.

Neisi et al. fabricated a nanocomposite, PANI/Cu-MOF, by a two-step procedure comprising the chemical polymerization of aniline monomer and Cu-MOFs at ambient temperature [105]. The composite electrode illustrates better capacitive characteristics compared with the bare Cu-MOF. In addition, the CV outcomes demonstrated that the PANI/Cu-MOF electrode possesses a superior specific capacitance (734 F g^−1^ at 5 mV s^−1^) with decent electrochemical cyclic stability.

Ternary MOF composite materials have attracted more attention compared to binary MOF-derived composite electrodes profiting from the synergetic effect of three different constituents [106]. Further inclusive properties are assembled by several components.

For example, Gong et al. prepared a multiporous (micro, meso, and macropores) architecture electrode material with three-dimensional porous carbon nanotubes sponges (porous CNTS) as a base surface for the successive incorporation of PANI and MOF [107]. The different pores-enriched architecture of the sponge favored the penetration of precursors as well as the uniform dispersion of PANI and MOF in the nanotubes. The multiporous architecture of CNTS not only offers a communication pathway for electrons but also provides networks for the rapid distribution of ions. The layered MOF provides an additional ion storage reservoir, while the MOFs are connected to the insulating PANI wires. In addition, the composite structure requires no mechanical binders or conductive additives and has excellent capacity combined with compressive, flexible, and moderately extraordinary specific capacitance.

The specific capacitance characteristic of CNTS was synergistically enhanced by the incorporation of ZIF-8, ZIF-67, and PANI. The specific capacitance value increased from 89 to 746 F g^−1^, and a highest specific energy of 28.9 Wh kg^−1^ was achieved. Furthermore, the prepared composite electrode was compressive, flexible, and has outstanding specific capacitance. Therefore, it could open new avenue for flexible energy storage devices.

**Figure 5:**
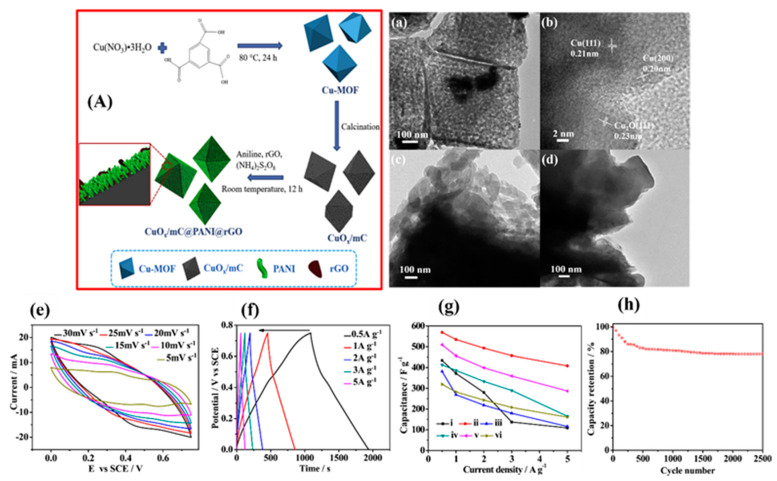
(**A**) Pictorial representation of the synthesis of CuO_x_@mC_700_@PANI@rGO composites; (**a**,**b**) represents the TEM and HR-TEM pictures of CuO_x_@mC_700_; (**c**,**d**) HR-TEM images of CuO_x_@mC_700_@PANI and CuO_x_@mC_700_@PANI@rGO, respectively; (**e**) CV curves of CuO_x_@mC_700_@PANI@rGO at different sweep rates; (**f**) GCD profiles of CuO_x_@mC_700_@PANI@rGO with various current densities; (**g**) comparative plot of the specific capacitance of different electrode materials versus current density variation; (**h**) cyclability of CuO_x_@mC_700_@PANI@rGO electrode at 2.0 A g^−1^. Reproduced with the permission from [108]. Copyright 2018 Elsevier.

He et al. prepared a multi-component hybrid of copper MOF-derived copper oxide@mesoporous carbon (CuO_x_@mC) entrenched with PANI and reduced graphene oxide (rGO) by in situ polymerization (CuO_x_@mC@PANI@rGO) [108]. The sequence of as-synthesized CuO_x_@mC@PANI@rGO composites was investigated for supercapacitor applications, and the schematic representation of the reaction protocol is depicted in Figure 5A.

Due to the ordered octahedral structure of CuO_x_@mC composites, a uniform and extremely well-organized interface layer of PANI with rGO nanosheets was formed on the surface of the CuO_x_@mC architecture. This effective conductive network could increase ion transport and redox behavior at the electrode/electrolyte interface, resulting in enhanced electrical conductivity and supercapacitor performances. TEM and HR-TEM images of CuO_x_@mC_700_, CuO_x_@mC_700_@PANI, and CuO_x_@mC_700_@PANI@rGO are presented in Figure 5a–d. From Figure 5a, we can see that the polyhedron crystals are approximately 500 nm in size, in which CuO_x_ particles are highly distributed in amorphous carbon. In the meantime, the HR-TEM image (Figure 5b) illustrated the distance of 0.20 and 0.21 nm, which can be indexed for the interplanar spacing between the cubic phase of Cu (200) and (111) planes, respectively. Furthermore, the CuO_x_@mC_700_@PANI exhibited a huge and uneven surface together with many nanowires on the external surface, as shown in Figure 5c.

Figure 5d demonstrates the stretchy and crumpled landscapes of rGO sheets which were examined after the incorporation of rGO nanosheets. The specific capacitance characteristics of the prepared composite electrode, CuO_x_@mC_700_@PANI@rGO, were further explored by continuous electrochemical measurements. Figure 5e demonstrates the cyclic voltammogram plots of CuO_x_@mC_700_@PANI@rGO at different sweep rates ranging between 5 and 30 mV s^−1^. With increasing the sweep rate, the current densities of the CV plots also increased. Nevertheless, the redox peaks shifted negatively and positively due to the electrode resistance [109].

Figure 5f displays the galvanostatic charge–discharge (GCD) plateaus in which typical triangular shapes were obtained at different densities of current, signifying excellent capacitance characteristic and reversibility. In addition, the specific capacitance decreases with raising the current density. By varying the pyrolysis temperature of Cu-MOF, the ternary CuO_x_@mC_700_@PANI@rGO, attained at 700 °C, displayed a superior specific capacitance of 534.5 F g^−1^ and extraordinary cyclability (Figure 5g). In contrast, the resulted CuO_x_@mC@PANI displayed a specific capacitance only of 456.0 F g^−1^ at 1 A g^−1^. Furthermore, it retains 70% of its initial capacitance over 2500 GCD curves (Figure 5h). This research has led to new insights into the study of metal oxide–carbon hybrids with morphologically controlled microstructures, where the beneficial function in the PANI is thought to be a hidden approach to improve the performance of these composites in supercapacitors.

Liu et al. prepared an electrode material, for supercapacitor applications, through the in situ formation of ZIF-8 onto the surface of ZnO followed by the deposition of thin PANI film (PANI/ZnO/ZIF-8/G/PC) [110]. The exceptional electrode architecture efficiently improved the performance of the supercapacitors. The assembled electrode, PANI/ZnO/ZIF-8/G/PC, exhibited a superior areal capacitance value of 1.378 F cm^−2^ at 1 mA cm^−1^ compared with the existing textile-based electrode materials (WO_3_/polyester/graphene and cotton/graphene). Furthermore, the authors constructed PANI/ZnO/ZIF-8/G/PC electrode in a flexible supercapacitor, where it delivered a good specific energy of 235 µWh cm^−3^ at a specific power of 1542 µW cm^−3^.

The composites of CPs with MOFs helped assemble highly efficient electrode materials, especially for PANI-based SCs. Nevertheless, such composite electrodes operate with Faradic redox reactions, which eventually decompose the electrolyte and shorten the lifetime of the supercapacitor device. To date, very few reports have investigated PANI/MOF-based electrode materials for supercapacitor applications [111]. This might be due to its comparatively inferior water stability and the wide distribution of most MOFs, which can create difficulties in identifying the appropriate preparation methodology for perceiving PANI/MOF composites. In addition to PANI, forthcoming inquiries on this topic may be driven by the process of various substitutes, such as PPy, PEDOT, and P3HT. The combination of such CPs with MOFs could lead to the arrangement of highly well-organized and flexible electrodes for high-performance supercapacitors [112,113]. Table 1 comprises various PANI/MOF-derived electrode materials for supercapacitor applications.

## 5. Conclusions and Future Perspectives

The demand for alternative energy resources and storage systems is increasing as conventional fossil fuels are gradually decreasing. Fossil fuels are sources of conventional energy production but have been gradually transitioned to the existing advanced technologies with a prominence of renewable resources such as solar, tidal, and wind. Despite consistent increases in energy prices, the customers’ needs are mounting rapidly due to an increase in populations, economic growth, per capita consumption, supply at remote places, and stationary forms for machines and portable electronics. The energy storage may allow the flexible generation and delivery of stable energy for meeting the demands of end users. The requirements for energy storage will triple the current values by 2050 where unique devices and systems are required. Protecting the ecology is an important effort related to the requirement of new technologies. Electrode material plays a major role in defining the practical viability of any energy storage device. For example, supercapacitors that can be used in practice should attain the technical needs of excellent specific capacity, specific energy, and specific power as well as long-term cyclability.

Briefly, the present review article describes the recent developments in electrode materials with their design, synthesis, and use of supercapacitors. PANI shows high specific capacitance value but displays a shorter life-cycle, whereas MOFs exhibit poor conductivity and specific capacitance. To overcome the shortcoming of PANI and enhance the conductivity and specific capacitance, PANI and MOF were composited.

There is no doubt that a wide range of PANI/MOFs and their derivatives have been well-initiated to catch enhancements in electrochemical behavior in recent years. Nevertheless, there are still numerous disputes and prospects for researchers/scientific communities to further investigate this interesting topic. The future of the PANI supercapacitor mainly relies on the adequate structure of the associated nanocomposites. However, a commercial supercapacitor is not based on a simple nanocomposite that mixes two composites; instead, a delicate structure is needed to place the MOF with the interacting surfaces between the polymer chains of PANI or vice versa. This design can take advantage of the flexibility of PANI for the development of flexible supercapacitors, which are in high demand. There are relatively minimal precursors or templates involving various MOFs (for example ZIF-8, ZIF-67, MOF-5, MOF-74, MIL-101) available to create an MOF and its derivative materials for supercapacitors. Even better starting materials and templates need to be created to attain new functional materials with unique architectures.

A wide cut-off voltage is frequently considered to be one of the crucial factors to enhance the supercapacitor performance. Still, the main barrier of the use of aqueous electrolyte is the dissociation of water that occurs when the voltage surpasses 1.23 V. PANI/MOFs as supercapacitor electrodes are still concerned with aqueous medium and the bench scale level. Therefore, in-depth research of solid-state supercapacitors (SSC) with a wide voltage window is required. Ecological, inexpensive, and high-yielding PANI/MOFs-based energy storage devices are expected to exist soon due to the development of PANI/MOFs technology.

In conclusion, the commercial usage of PANI/MOFs supercapacitors are still at the laboratory stage. Additional inputs in this vast research field will promote the expansion of PANI/MOF research into the next-generation environment-friendly energy storage systems. As this research area has seen tremendous growth, we can assume the development of highly efficient energy storage devices with the forthcoming developments in pilot-scale machineries of PANI/MOFs.

## Figures and Tables

**Table 1:** PANI/MOF composite-based electrode materials for supercapacitor applications.

S. No.	Electrode Materials	Specific Capacitance		Electrolyte	Specific Energy	Specific Power	Cyclability/ Capacitance Retention	Ref.
		3-ES	2-ES					
1	PANI-ZIF-67/CC	2146 mF cm^−2^ @ 10 mV s^−1^	SSC: 35 mF cm^−2^	3-ES: 3 M KCl; SSC: H_2_SO_4_/PVA	0.0161 mWh cm^−3^	0.833 W cm^−3^	2000 GCD cycles; 80%	[93]
2	Co_3_S_4_/PANI	106 F g^−1^ @ 1 A g^−1^	ASC: 114.6 F g^−1^ @ 1 A g^−1^	3-ES and ASC: 6 M KOH	40.75 Wh kg^−1^	800 W kg^−1^	20,000 GCD cycles; 88%	[94]
3	MOF/PANI	162.5 C g^−1^ @ 0.4 A g^−1^	ASC: 104.5 C g^−1^ @ 1 A g^−1^	3-ES and ASC: 1 M KOH	23.2 Wh kg^−1^	1600 W kg^−1^	3000 GCD cycles; 146%	[95]
4	PC-MOFs/PANI	534.16 F g^−1^ @ 0.2 A g^−1^	SSC: 140 F g^−1^ @ 0.2 A g^−1^	SSC: H_2_SO_4_/PVA	9.72 μWh cm^−2^	199.99 μW cm^−2^	10,000 cycles; 94.4%	[96]
5	MOF/PANI	1100 F g^−1^@ 1 mV s^−1^	SSC: 236 140 F g^−1^ @ 1 A g^−1^	1 M H_2_SO_4_	21 Wh kg^−1^	400 W kg^−1^	20,000 GCD cycles; 86%	[97]
6	PANI/Fe-BTC	346 F g^−1^ @ 20 mV s^−1^	---	0.5 M H_2_SO_4_	---	---	---	[98]
7	MOF/PANI	719.2 mF cm^−2^ @ 0.5 mA cm^−2^	MSCs: 528.5 mF cm^−2^ @ 10 mA cm^−2^	MSCs: H_2_SO_4_/PVA	443.7 mW cm^−3^	3218.4 μW cm^−2^	6000 GCD cycles; 87.6%	[99]
8	Zn-MOF/PANI	477 F g^−1^ @ 1 A g^−1^	---	1 M H_2_SO_4_	---	---	---	[100]
9	PANI/UiO-66	1015 F g^−1^ @ 1 A g^−1^	SSC: 647 F g^−1^ @ 1 A g^−1^	H_2_SO_4_/PVA	78.8 Wh kg^−1^	200 W kg^−1^	5000 GCD cycles; 91%	[101]
10	ZIF-67@PANI	2497 F g^−1^ @ 1 A g^−1^	SSC: 512 F g^−1^ @ 1 A g^−1^	KOH	71.1 Wh kg^−1^	504.72 W kg^−1^	9000 GCD cycles; 92.3%	[102]
11	CFP/ZIF-L/PANI	681 mF cm^−2^ @ 1 mA cm^−2^	---	3 M KCl	---	---	3000 GCD cycles; 82.6%	[103]
12	ZIF-8/PANI	395.4 F g^−1^ @ 0.2 A g^−1^	ASC: 28.1 mF cm^−2^ @ 0.1 mA cm^−2^	1 M H_2_SO_4_	3.2 μWh cm^−2^	1.1 mW cm^−2^	1000 GCD cycles; 78.4%	[104]
13	PANI/Cu-MOF	734 F g^−1^ @ 5 mV s^−1^	---	6 M KOH	---	---	4000 GCD cycles; 98%	[105]
14	CNT/MOF/PANI	342.5 F g^−1^ @ 1 A g^−1^	---	---	28.9 Wh kg^−1^	~800 W kg^−1^	---	[107]
15	CuO_x_@mC@PANI@rGO	534.5 F g^−1^@ 1 A g^−1^	---	1 M H_2_SO_4_	---	---	2500 GCD cycles; 80%	[108]
16	PANI/ZnO/ZIF-8/G/PC	1.378 F cm^−2^ @ 1 mA cm^−1^	SSC: ---	SSC: H_2_SO_4_/PVA	235 μWh cm^−3^	1542 μW cm^−3^	---	[110]

Note: 3-ES: Three-electrode system; 2-ES: Two-electrode system; KCl: Potassium chloride; CNT: Carbon nanotube; PC: Porous carbon; CFP: Carbon fiber paper; SSC: Symmetric supercapacitor; ASC: Asymmetric supercapacitor; MSCs: Micro-supercapacitors; KOH: Potassium hydroxide; H_2_SO_4_: Sulfuric acid; PVA: Polyvinyl alcohol.

## Data Availability

No new data were created or analyzed in this study. Data sharing is not applicable to this article.

## Supplementary Materials

The following supporting information can be downloaded at: https://www.mdpi.com/article/10.3390/nano12091511/s1, Table S1: Specific energy and specific power of recently reported articles in supercapacitors. References [6,114,115,116,117,118,119,120,121,122] are cited in the supplementary materials.
