# Supplementary material for: Recent Advancements of Polyaniline/Metal Organic Framework (PANI/MOF) Composite Electrodes for Supercapacitor Applications: A Critical Review
Source: Nanomaterials (Basel). 2022 Apr 29;12(9):1511. doi: 10.3390/nano12091511 (PMC9105330; doi:10.3390/nano12091511)
Supplement: Supplementary file 1 [file nanomaterials-12-01511-s001.zip › nanomaterials-1657492-supplementary.pdf]

nanomaterials

MDPI

Supplementary Materials

Recent Advancements of Polyaniline/Metal Organic Framework (PANI/MOF) Composite Electrodes for Supercapacitor Applications: A Critical Review

Rajangam Vinodh [1,†], Rajendran Suresh Babu [2,†], Sangaraju Sambasivam [3,†], Chandu V. V. Muralee Gopi [4], Salem Alzahmi [5,7,*], Hee-Je Kim [6,*], Ana Lucia Ferreira de Barros [2] and Ihab M. Obaidat [3,7,*]

Table S1. Specific energy and specific power of recently reported articles in supercapacitors.

Electrode Material	Specific energy	Specific power	Ref.
NiAl-LDHs//AC	21	700	[114]
CoAl LDHCNTs//AC	28	444.1	[115]
NiO nanoflakes//AC	52.4	32000	[116]
Porous carbon derived from used baby diaper	9.4	1542	[117]
HCP of [P(DVB:PVC)] derived porous carbon	39.47	699.96	[6]
P-$Co_{0.21}Ni_{0.79}MoO_4$//AC	49.2	747.7	[118]
VS_2//AC	42	700	[119]
$NiMoO_4$//AC	18	704	[120]
Chitin-derived hierarchically porous carbon microspheres/polyaniline	8.9	1644	[121]
MnO_2–graphene–chitosan composite	80.40	2894.61	[122]

References

6. Kim, I.; Vinodh, R.; Gopi, C.V.V.M.; Kim, H.-J.; Babu, R.S.; Deviprasath, C.; Devendiran, M.; Kim, S. Novel porous carbon electrode derived from hypercross-linked polymer of poly(divinylbenzene-co-vinyl benzyl chloride) for supercapacitor applications. *J. Energy Storage* **2021**, *43*, 103287.
114. Zhang, L.; Yao, H.; Li, Z.; Sun, P.; Liu, F.; Dong, C.; Wang, J.; Li, Z.; Wu, M.; Zhang, C.; et al. Synthesis of delaminated layered double hydroxides and their assembly with graphene oxide for supercapacitor application. *J. Alloys Compd.* **2017**, *711*, 31–41.
115. Yu, L.; Shi, N.; Liu, Q.; Wang, J.; Yang, B.; Wang, B.; Yan, H.; Sun, Y.; Jing, X. Facile synthesis of exfoliated Co–Al LDH–carbon nanotube composites with high performance as supercapacitor electrodes. *Phys. Chem. Chem. Phys.* **2014**, *16*, 17936–17942, https://doi.org/10.1039/c4cp02020k.
116. Vinodh, R.; Babu, R.S.; Atchudan, R.; Kim, H.-J.; Yi, M.; Samyn, L.M.; de Barros, A.L.F. Fabrication of High-Performance Asymmetric Supercapacitor Consists of Nickel Oxide and Activated Carbon (NiO//AC). *Catalysts* **2022**, *12*, 375, https://doi.org/10.3390/catal12040375.
117. Atchudan, R.; Edison, T.N.J.I.; Perumal, S.; Thirukumaran, P.; Vinodh, R.; Lee, Y.R. Green synthesis of nitrogen-doped carbon nanograss for supercapacitors. *J. Taiwan Inst. Chem. Eng.* **2019**, *102*, 475–486, https://doi.org/10.1016/j.jtice.2019.06.020.
118. Xing, T.; Ouyang, Y.; Chen, Y.; Zheng, L.; Wu, C.; Wang, X. P-doped ternary transition metal oxide as electrode material of asymmetric supercapacitor. *J. Energy Storage* **2020**, *28*, 101248, https://doi.org/10.1016/j.est.2020.101248.
119. Masikhwa, T.M.; Barzegar, F.; Dangbegnon, J.K.; Bello, A.; Madito, M.J.; Momodu, D.; Manyala, N. Asymmetric supercapacitor based on VS_2 nanosheets and activated carbon materials. *RSC Adv.* **2016**, *6*, 38990–39000, https://doi.org/10.1039/c5ra27155j.
120. Neeraj, N.S.; Mordina, B.; Srivastava, A.K.; Mukhopadhyay, K.; Prasad, N.E. Impact of process condi-tions on the electrochemical performances of $NiMoO_4$ nanorods and activated carbon based asymmetric supercapacitor. *Appl. Surf. Sci.*, **2019**, *473*, 807–819.
121. Gao, L.; Xiong, L.; Xu, D.; Cai, J.; Huang, L.; Zhou, J.; Zhang, L. Distinctive Construction of Chitin-Derived Hierarchically Porous Carbon Microspheres/Polyaniline for High-Rate Supercapacitors. *ACS Appl. Mater. Interfaces* **2018**, *10*, 28918–28927.
122. Salleh, N.A.; Kheawhom, S.; Mohamad, A.A. Chitosan as biopolymer binder for graphene in superca-pacitor electrode. *Results Phys.* **2021**, *25*, 104244.
